# A reclassification of the millipede superfamily Trichopolydesmoidea, with descriptions of two new species from the Aegean region (Diplopoda, Polydesmida)

**DOI:** 10.3897/zookeys.340.6295

**Published:** 2013-10-04

**Authors:** Sergei I. Golovatch

**Affiliations:** 1 Institute for Problems of Ecology and Evolution, Russian Academy of Sciences, Moscow, Russia

**Keywords:** Diplopoda, Trichopolydesmidae, Fuhrmannodesmidae, taxonomy, new species, cave, Greece

## Abstract

Two new species are described from caves in several Greek islands in the Aegean Sea: *Galliocookia gracilis* **sp. n.**, a presumed troglobite from Rhodes, Dodecanese Islands, and *Sphaeroparia simplex* **sp. n.**, likely a troglophile from Kithnos, Cyclades, and Chios, Eastern Sporades. These genera are assigned to the family Trichopolydesmidae Verhoeff, 1910. Because *Sphaeroparia* Attems, 1909, an Afrotropical genus, nicely bridges the gap, both morphologically and geographically, between the Euro-Mediterranean Trichopolydesmidae and the much more diverse, pantropical Fuhrmannodesmidae Brölemann, 1916, the latter family is considered as a new junior subjective synonym of the former, **syn. n.** Thus expanded, the family Trichopolydesmidae is rediagnosed and its position within the superfamily Trichopolydesmoidea refined. Because the families Mastigonodesmidae Attems, 1914, Macrosternodesmidae Brölemann, 1916 and Nearctodesmidae Chamberlin & Hoffman, 1950 are also formally synonymized with Trichopolydesmidae, **syn. n.**, the Trichopolydesmoidea currently contains only two families, Trichopolydesmidae and Opisotretidae Hoffman, 1980.

## Introduction

According to Shear (2011), the millipede superfamily Trichopolydesmoidea includes only three families: Fuhrmannodesmidae Brölemann, 1916, Macrosternodesmidae Brölemann, 1916 and Nearctodesmidae Chamberlin & Hoffman, 1958. Strangely enough, the nominotypical family Trichopolydesmidae Verhoeff, 1910 was omitted. More recently, Golovatch et al. (2013) have added therein not only the Trichopolydesmidae, but also the Opisotretidae Hoffman, 1980, the latter family previously (Simonsen 1990, Shear 2011) considered as representing a superfamily of its own, Opisotretoidea.

Prompted by the discovery of two new species of trichopolydesmoids in caves on three Aegean islands of Greece, this superfamily is reclassified. One of the genera appears to so nicely bridge the gap between the Euro-Mediterranean Trichopolydesmidae and the much more diverse, pantropical Fuhrmannodesmidae, that the latter family is formally synonymized here with the former.

## Material and methods

The material treated below had been taken in 1987 by Petar Beron, of the National Museum of Natural History, Sofia, Bulgaria (NMNHS), in caves on a few Aegean islands of Greece and was offered to me for treatment in August 2013, when I visited the NMNHS in the framework of the Russian-Bulgarian interacademician exchange programme. Most of the type material will be returned to the NMNHS, with only two paratypes retained for the collection of the Zoological Museum, State University of Moscow, Russia (ZMUM).

## Descriptions

### Galliocookia gracilis sp. n.

http://zoobank.org/97419B2E-E237-4A96-A507-2A9FBF4ACFC0

http://species-id.net/wiki/Galliocookia_gracilis

Figs 1 –6

#### Type material.

Holotype ♂ (NMNHS), Greece, Rhodes Island, village Archangelos, Cave Coumellos, 02.05.1987, leg. P. Beron.

Paratypes: 1 ♂, 1 ♀, 1 ♂ subadult (NMNHS), same locality, together with holotype.

#### Diagnosis.

Differs from the other species of *Galliocookia* Ribaut, 1955, all three from caves in southern France (Mauriès 1983), by the somewhat larger size (8-9 mm versus maximum 6.7 mm in ♀ *Galliocookia balazuci* Mauriès, 1983, 7.5 mm in ♀ *Galliocookia fagei* Ribaut, 1955 or 7.7 mm in ♀ *Galliocookia leclerci* Mauriès, 1983), the presence of a distodorsal field of sensilla also in antennomere 5, of porosteles, the peculiarly upturned pre-apical teeth flanking the epiproct, as well as a trifid, not bifid, tip of the gonopod (with a small stump proximal to the solenomere).

#### Name.

To emphasize the highly gracile looks of this species.

#### Description.

**Figures 1, 2.**
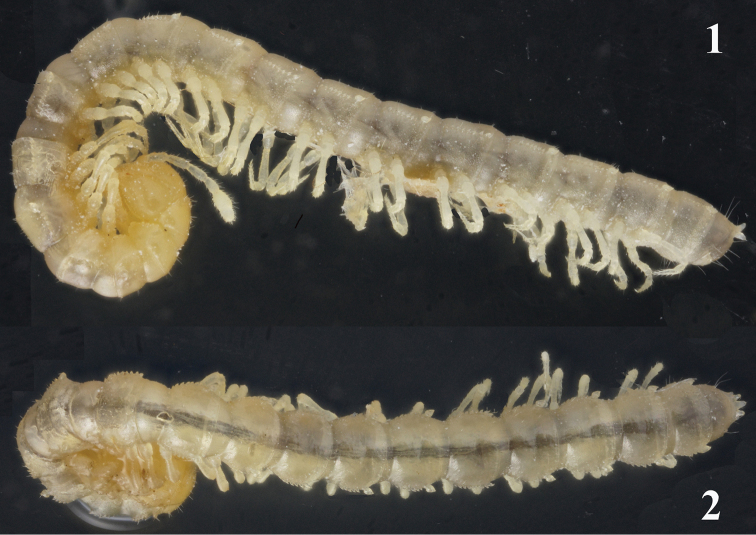
Habitus of *Galliocookia gracilis* sp. n., holotype, lateral and dorsal views, respectively. Photos by K. Makarov, not taken to scale.

Length of adults ca 8.0 (♂) or 9.0 mm (♀), width of midbody pro- and metazona 0.8 and 0.9 mm (♂, ♀), respectively. Coloration in alcohol uniformly pallid to, in front body quarter, light yellowish (Figs 1, 2).

**Figures 3–6.**
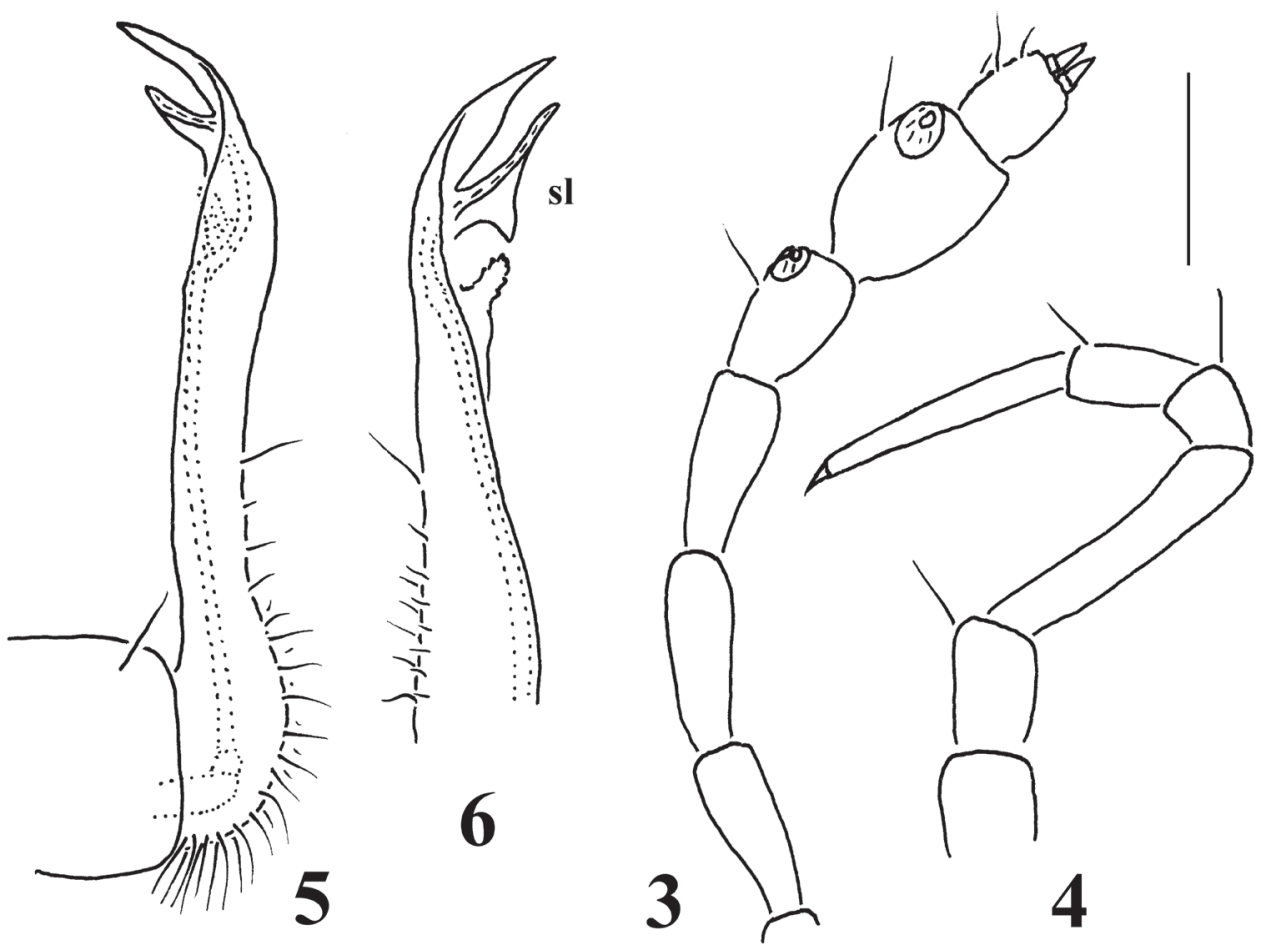
*Galliocookia gracilis* sp. n., holotype **3** antenna, lateral view **4** leg 9, **5, 6** right and left gonopods, lateral and sublateral views, respectively. Scale bars: **3, 4** 0.2 mm; **5, 6** 0.4 mm.

Body with 19 (♂) or 20 (♀) segments, strongly moniliform. Tegument smooth, mainly slightly shining, texture very delicately alveolate. Head densely pilose throughout; ♂ epicranial modifications absent, frons being regularly convex in both sexes. Antennae long and slender, reaching behind segment 2 when stretched dorsally (♂, ♀); antennomeres 2-4 and 6 subequal in length, but 6^th^ clearly the highest (height being measured from ventral to dorsal margin) (Figs 1, 3); antennomeres 5 and 6 each with a distinct, round, distodorsal, compact field of sensilla.

In width, head = segments 6-18(19) > 5 > 4 > 2 = 3 > collum; body suddenly tapering on telson. Collum ellipsoid, acutangular caudolaterally, like most of following metaterga with three transverse, rather regular rows of setae until segment 15 or 16, following metaterga with 3-4 less regular rows in front of a 4^th^ or 5^th^ regular row at caudal margin. Tergal setae very short, simple, sharp, only about 1/5-1/6 the length of a metatergum, a little longer only on collum and penultimate segment, mostly 3+3 in each row, but gradually growing to about 4+4 or 5+5 per row towards telson. A very faint transverse sulcus in caudal 1/3 of metaterga in front of caudal row of setae. Dorsum invariably convex. Paraterga poorly developed, especially so in ♀, visible starting from collum, invariably slightly declivous, set rather high (mainly at about upper quarter or third of midbody height in ♂ and ♀, respectively), slightly, but regularly rounded laterally; lateral margin of postcollum paraterga clearly serrate, with 6-7 subequal, often setigerous indentations in front of a somewhat (♀) or much (♂) larger, isolated, sometimes nearly pointed tubercle, this in pore-bearing segments turning into a conspicuous porostele (Figs 1, 2). This caudal tubercle or porostele of paraterga drawn caudolaterad, but never extending behind rear tergal margin. Pore formula normal: 5, 7, 9, 10, 12, 13, 15-18(19), ozopores round, dorsal, quite evident due to their porosteles. Stricture between pro- and metazona wide, shallow and smooth. Limbus very finely microspiculate. Epiproct conical, rather long, but barely extending behind 2+2 conspicuous, pre-apical teeth, caudalmost of which unusually strong and upturned (Figs 1, 2). Hypoproct trapeziform.

Sterna clearly separated, unmodified. Legs rather long and slender, without modified setae, ca 1.3–1.4 (♂) or 1.1–1.2 (♀) times as long as midbody height, femora and tarsi longest, claw short (Fig. 4). Epigynal ridge very low, inconspicuous.

Gonopod aperture transversely oblong-oval, taking up most of ventral part of metazonite 7. Gonopods (Figs 5, 6) with small, subglobose, evidently exposed, medially fused coxae, each carrying only 1 long seta distolaterally and a long, curved cannula distomesally; a scaly texture on lateral face absent. Telopodite suberect, slender and long, up to about its half being taken up by an elongate, setose prefemoral part; acropodite slightly helicoid, its apical lobe subacuminate, with a pre-apical, lobe-shaped, subtriangular solenomere (**sl**) and a short, papillate, rounded stump a little proximally. Both prefemoral part and acropodite strictly coaxial, in situ directed forward and parallel to each other. No traces of accessory seminal chamber or hairy pulvillus.

#### Remarks.

Among the Euro-Mediterranean genera of Trichopolydesmoidea, only some show a deeply bipartite and strongly curved gonopod telopodite, the prefemoral part of which is quite elongate, but lies more or less transversely to strongly angular, largely (sub)parallel telopodites and extends across the nearly entire ventral width of segment 7. Such are *Trichopolydesmus* Verhoeff, 1898 (together with *Banatodesmus* Tabacaru, 1980), *Bacillidesmus* Attems, 1898, *Napocodesmus* Ceuca, 1974 and *Caucasodesmus* Golovatch, 1985. In contrast, the gonotelopodites in *Verhoeffodesmus* Strasser, 1959, *Cottodesmus* Verhoeff, 1936 and *Occitanocookia* Mauriès, 1980 have increasingly shortened prefemoral parts, being enlarged and laterally flattened distad, unipartite and mostly less strongly curved, in *Cottodesmus* and *Occitanocookia* also devoid of a solenomere, but sometimes supplied instead with what can be seen as a primordial accessory seminal chamber. In *Trichopolydesmus*, *Heterocookia* Silvestri, 1898, *Ingurtidorgius* Strasser, 1974 and, especially, *Mastigonodesmus* Silvestri, 1898, the solenomere is flagelliform, branching off near the base of the femorite. In all these genera, the gonotelopodites are strongly exposed, not sunken inside an obvious central coxal cavity (= gonocoel). A modest gonocoel seems to only be observed in *Ingurtidorgius* and *Haplocookia* Brölemann, 1915. This latter genus does resemble *Galliocookia*, but its gonopod telopodite is clearly curved, there is a small gonocoel and both sexes have 20 body segments (see also review by Mauriès 1983).

The new species definitely belongs to *Galliocookia*, sharing with the other three congeners (see Diagnosis above) a small coxa and a slender, simple and suberect telopodite, the latter showing an extended prefemoral part strictly coaxial with the acropodite. Moreover, the solenomere is likewise modest, lobe-shaped and located distally. Even the presence of 19 or 20 body segments in the male and female, respectively, as well as of a normal ozopore formula, setose metaterga and laterally serrate/indentate paraterga coincide.

Biogeographically, the discovery of a *Galliocookia* in the Aegean region, so far away from France, is remarkable, emphasizing a pan-Mediterranean distribution pattern of this currently purely cavernicolous, likely troglobitic genus.

### Sphaeroparia simplex sp. n.

http://zoobank.org/795A5831-ADEA-49EC-977F-AAE9878037B2

http://species-id.net/wiki/Sphaeroparia_simplex

Figs 7 –17

#### Type material.

Holotype ♂ (NMNHS), Greece, Kithnos Island, village Dryopis near Phirès, Cave Katafyki, 08.05.1987, leg. P. Beron.

Paratypes: 1 ♂, 4 ♀♀, 1 ♂ subadult (19 body segments), 2 fragments (NMNHS) + 1 ♂, 1 ♀ (ZMUM), same locality, together with holotype; 1 ♂, 1 ♀, 1 ♀ fragment (head and first 12 body segments) (NMNHS), Greece, Chios Island, village Haghios Galos (Agiongalas, Haghia Gala), 65 km from town of Chios, Cave Hagiogalousaina, 12.05.1987, leg. P. Beron.

#### Diagnosis.

Differs from the other 30+ species of *Sphaeroparia* Attems, 1909, many of which have been reviewed by Mauriès and Heymer (1996), by 20 body segments in both sexes and the presence of an axial sternal process on the especially simple gonopods, including a nearly fully suppressed solenomere.

#### Name.

To emphasize the highly simple gonopods in this species.

#### Description.

**Figures 7–8.**
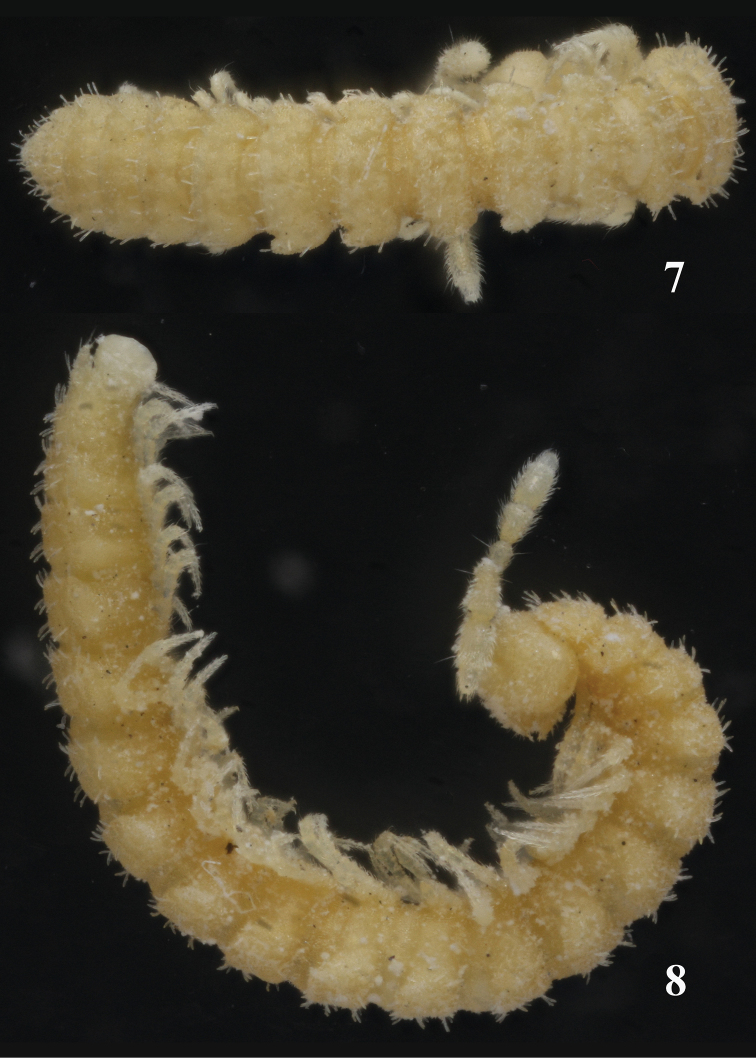
Habitus of *Sphaeroparia simplex* sp. n., holotype, dorsal and lateral views, respectively. Photos by K. Makarov, not taken to scale.

**Figures 9, 10.**
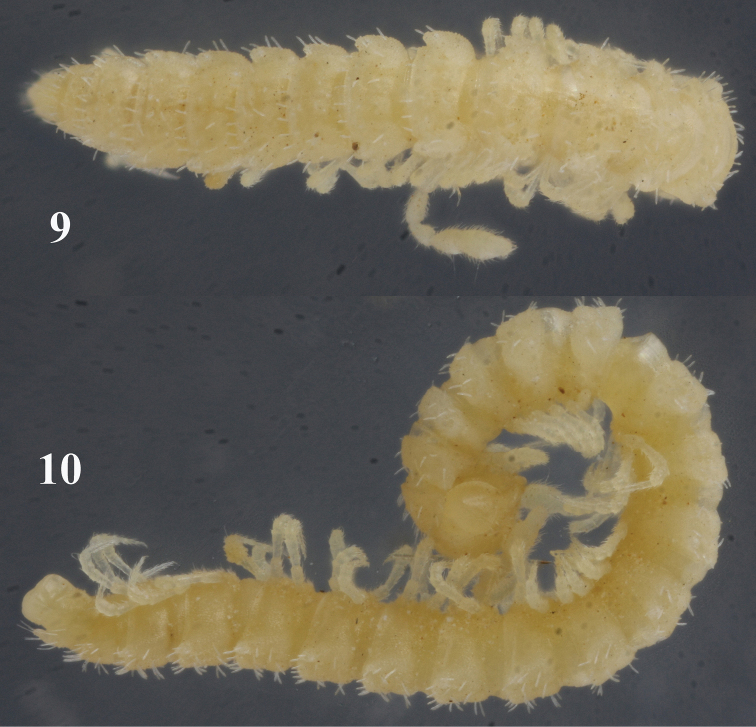
Habitus of *Sphaeroparia simplex* sp. n., ♂ paratype from Kithnos, dorsal and lateral views, respectively. Photos by K. Makarov, not taken to scale.

Length of adults ca 5.0–5.5 (♂) or 6.0–7.0 mm (♀), width of midbody pro- and metazona 0.55 and 0.7 mm (♂, ♀), respectively. Coloration in alcohol uniformly pallid to light yellowish (Figs 7–10).

**Figures 11–14.**
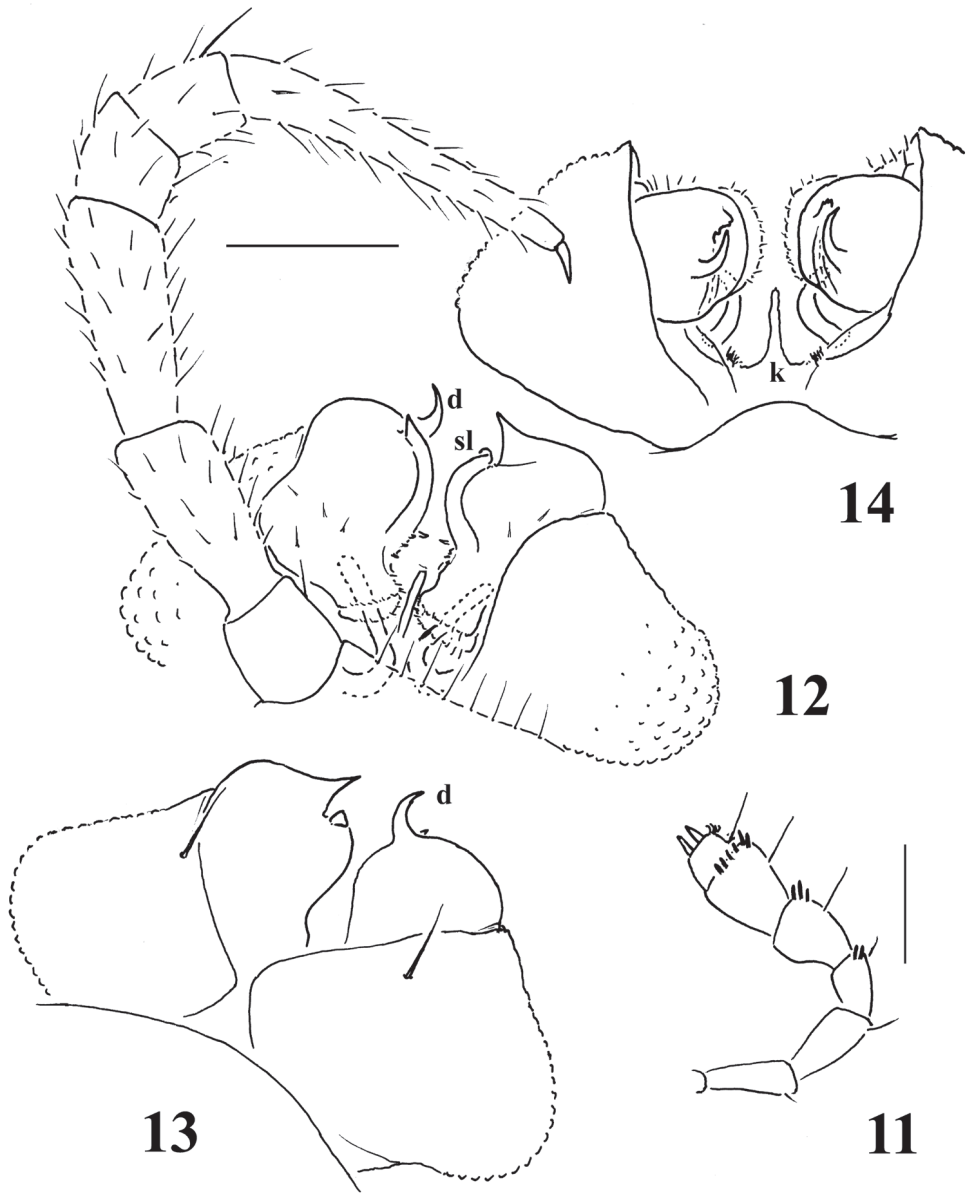
*Sphaeroparia simplex* sp. n., holotype (**12, 13**) and ♂ paratype (**11, 14**) from Kithnos **11** antenna, lateral view **12** leg 9 and both gonopods, caudal view **13, 14** both gonopods, oral and ventral views, respectively. Scale bars: **11** 0.2 mm; **12–14** 0.1 mm.

**Figures 15–17.**
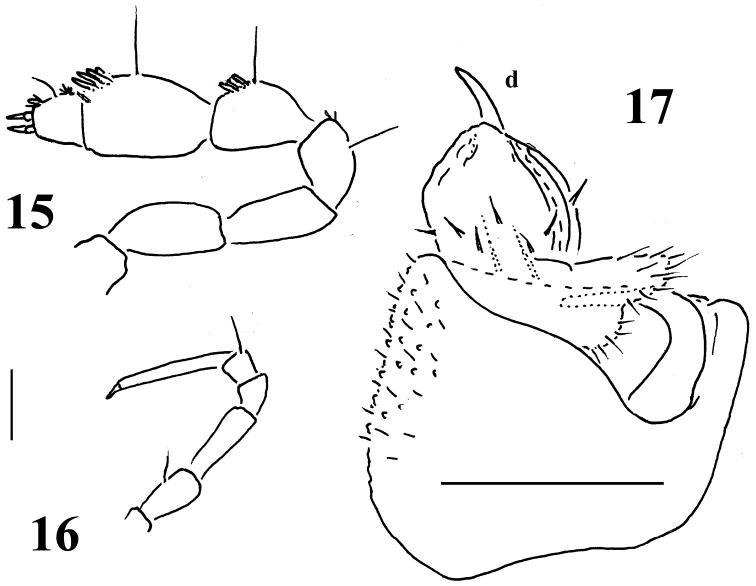
*Sphaeroparia simplex* sp. n., ♂ paratype from Chios **15** antenna, lateral view **16** midbody leg **17** left gonopod, mesal view. Scale bars: 0.1 mm.

Body with 20 segments in both sexes. Tegument generally smooth, dull, texture very delicately alveolate. Head densely pilose throughout; ♂ epicranial modifications absent, frons being regularly convex in both sexes. Antennae rather short and clavate, nearly reaching end of segment 2 (♂) or collum (♀) when stretched dorsally; antennomeres 2, 3, 5 and 6 subequal in length, but both 5^th^ and 6^th^ clearly the highest (height being measured from ventral to dorsal side) (Figs 11, 15); antennomeres 4-6 each with a loose, indistinct, distodorsal group of increasingly long and numerous sensilla.

In width, head = segments 6-16(17) > 5 > 4 > 2 = 3 > collum; starting from segment 17, body gradually tapering towards telson. Collum ellipsoid, lobuliform caudolaterally, like most of following metaterga with 3 transverse, rather regular rows of 3+3 setae on minute knobs until segment 16, of 4+4 setae in segments 17(18)-19. Tergal setae medium-sized, slender, bacilliform, mostly about 1/4 the length of a metatergum, a little longer only on collum and penultimate segment. Usually a very faint transverse sulcus between first 2 rows of setae. Dorsum invariably convex. Paraterga poorly developed, especially so in ♀, visible starting from collum, invariably slightly declivous, set rather high (mainly at about upper third of midbody height in both sexes), slightly, but regularly rounded laterally; lateral margin of postcollum paraterga very poorly indentate, on each side usually with 3 subequal setigerous indentations in front of a rounded caudolateral lobule, the latter increasingly well drawn caudad, but never extending behind rear tergal margin (Figs 7–10). Pore formula normal: 5, 7, 9, 10, 12, 13, 15-18(19), ozopores round, dorsal, rather indistinct, lying at base of caudolateral lobule near lateral margin. Stricture between pro- and metazona wide, shallow and smooth. Limbus very finely microspiculate. Epiproct conical, rather long. Hypoproct trapeziform.

Sterna clearly separated, unmodified. Legs rather long and slender (Figs 8, 10, 12, 16), without modified setae, ca 1.2–1.3 (♂) or 1.0–1.1 (♀) times as long as midbody height, tarsi longest, claw short. Epigynal ridge very low, inconspicuous.

Gonopod aperture evident, transversely oblong-oval, taking up most of ventral part of metazonite 7. Gonopods (Figs 12–14, 17) with large, transversely subglobose, evidently exposed, medially fused, medially clearly excavate coxites, each microgranular/scaly and micropilose laterally, carrying only 1 long seta distofrontally and a long, curved cannula distomesally; sternum plate-like, with a slender, central, not too strongly chitinized process (**k**) between coxites. Telopodites short, stout, subglobose, sac-shaped, very simple, only moderately exposed below coxites, deeply sunken inside a prominent gonocoel; prefemoral part as usual, setose, quite elongate, about as long as, but clearly set at an angle to, acropodite, the latter with a distinct seminal groove running entirely on mesal side and terminating on a vestigial lobuliform solenomere (**sl**) at base of a rather strong, only faintly curved, apical spine (**d**). Neither an accessory seminal chamber nor a hairy pulvillus.

#### Remarks.

This species seems to be only a troglophile, which occurs in caves on two remote islands in the Aegean Sea. On the other hand, Cave Katafyki is known to support at least two presumed troglobites, i.e. the woodlouse *Cordioniscus kithnosi* Andreev, 1986 (Isopoda, Oniscidea, Styloniscidae) (Schmalfuss 2003) and the millipede *Syrioiulus andreevi* Mauriès, 1984 (Diplopoda, Julida, Julidae) (Mauriès 1984), while Cave Hagiogalousaina hosts the presumed troglobitic false-scorpion *Chthonius chius* Schawaller, 1990 (Pseudoscorpiones, Chthoniidae) (Harvey 2008) and the millipede *Hyleoglomeris subreducta* Golovatch, 2013 (Diplopoda, Glomerida, Glomeridae) (Golovatch in press).

The discovery of a *Sphaeroparia* in the Aegean region is even more remarkable than that of a *Galliocookia*. Indeed, *Sphaeroparia* is a rather large genus hitherto believed to be strictly Afrotropical, ranging from Liberia and Benin in western Africa, through Gabon and Zaire, to Kenya and Tanzania in eastern Africa (see review by Mauriès and Heymer 1996). Furthermore, it has been assigned to the large pantropical family Fuhrmannodesmidae (Hoffman 1980, Mauriès and Heymer 1996), as opposed to the Holarctic Macrosternodesmidae, the Nearctic Nearctodesmidae (often referred to as only a subfamily of Macrosternodesmidae), and the Euro-Mediterranean Trichopolydesmidae. The Fuhrmannodesmidae has long been considered as an artificial, composite group (e.g. Hoffman 1980, Mauriès and Heymer 1996, Golovatch et al. 2013), being distinguished from the above allies solely by its being tropical.

Golovatch (1994) provided an evolutionary scenario for the genera of Fuhrmannodesmidae known from South America, accepting as the basalmost those genera showing rather small, subglobose gonopod coxae that form no significant gonocoel in which to hinge the largely exposed, usually rather simple and elongate telopodites. Moreover, as in some true Trichopolydesmidae (see above), the prefemoral (= setose) part of the gonopod is mostly orientated transversely to the body axis, extending mesally across the entire width of the coxae. Following a series of transitional states, such forms ultimately culminate in having the gonopod coxae strongly enlarged, forming a large gonocoel in which to conceal the clearly shortened, usually highly complex and deeply sunken telopodites. Their prefemoral parts already tend to be positioned increasingly parallel to the body’s main axis, thus providing a transition between the usually small-sized Trichopolydesmoidea (= so-called “micropolydesmoids”) to the normally medium- to large-sized Polydesmoidea (= so-called “macropolydesmoids”).

Naturally, similar general trends can be surmised to have occurred in the fuhrmannodesmids of Central America and the Afrotropical and Oriental realms, which also support fairly diverse faunas of this family. Remarkably, none of the genera of Trichopolydesmidae (see above), all confined to Europe and the Mediterranean, though demonstrating a certain degree of variation in the length and orientation of the gonopod prefemoral part, has a deep gonocoel. Furthermore, *Galliocookia* as one of the few genera where the gonocoxae are particularly small while the prefemoral part is still elongate, but already strictly coaxial with a very strongly exposed acropodite represents an evolutionary extreme, apparently the basalmost situation. The discovery of a *Sphaeroparia* in the eastern Mediterranean, of an Afrotropical genus demonstrating a very large and deep gonocoel for the small and only poorly exposed telopodites to be hinged into, clearly represents the opposite, evolutionarily obviously the most advanced extreme. Therefore, the Euro-Mediterranean Trichopolydesmoidea appear to show basically the same full range of evolutionary trends in the development of gonopod structures as do at least the properly assessed South American representatives now assigned to Fuhrmannodesmidae! Among the Afrotropical Trichopolydesmoidea, *Sphaeroparia* is certainly the most speciose genus and Mauriès and Heymer (1996) divide it into as many as six subgenera, most of which they themselves admit to be ill-grounded. A *Sphaeroparia* species is likely to be present also in the Seychelles (Golovatch and Gerlach 2010). In contrast to the Euro-Mediterranean region, the Afrotropical realm is thus dominated by trichopolydesmoids showing a deep gonocoel between strongly hypertrophied gonopod coxae. Only *Peronorchus parvicollis* Attems, 1907, the type and still sole species of *Peronorchus* Attems, 1907, shows a far more basal gonopod structure quite comparable to that of *Trichopolydesmus*. That species was originally described from near Buitenzorg (= Bogor), Java, Indonesia, and it has since been redescribed from material from Mauritius, Indian Ocean and formally assigned to the family Trichopolydesmidae (Mauriès and Geoffroy 1999). However, *Peronorchus* has recently been transferred to Fuhrmannodesmidae (Golovatch et al. 2013).

Considering the above new evidence, I no longer hesitate to formally synonymize the family Fuhrmannodesmidae Brölemann, 1916 under Trichopolydesmidae Verhoeff, 1910, syn. n.

### A reclassification

The following refined classification and diagnosis of the superfamily Trichopolydesmoidea, as well as of its two constituent families can be proposed (see also Golovatch 2011, Golovatch et al. 2013).

#### Superfamily Trichopolydesmoidea

Verhoeff, 1910

##### Diagnosis.

Body largely polydesmoid, only 2–16 mm long, exceptionally up to 30 mm, usually ≤ 10 mm long, with 18–20 segments. Antennomeres 2-6 usually subequal in length, but 6^th^ normally highest; 5^th^ often, 6^th^ only exceptionally, devoid of an apicodorsal field of sensilla. ♂ head sometimes with vertigial modifications (humps, fossae etc.). Paraterga at most with only thin calluses, often devoid of these, lateral margin entire, only exceptionally lobulate (*Trilobodesmus* Golovatch & Mauriès, 2007), typically at least faintly incised and setigerous; metaterga devoid of a cerategument, more often with 3-4, more or less regular, transverse rows of low, polygonal or rounded, setigerous bosses, but rather usually these latter either obliterated or pronounced and more numerous. Ozopores flush, opening on dorsal surface of paraterga to be located at their lateral margin, pore formula normal. Sphaerotrichomes rather often present, usually affecting both tibiae and tarsi.

Gonopod aperture large, transversely ovoid, not extending onto prozonite, but sometimes spreading caudad between coxae 9; gonocoxae subglobose, sometimes with a distolateral projection; prefemoral parts usually lying transversely to main body axis and, together with coxae, taking up most of ventral extent of ♂ ring 7, quite often with an obvious gonocoel formed by enlarged coxae; cannula and seminal groove nearly always present, both absent only exceptionally; entire telopodites or, more usually, acropodites distal to prefemoral parts usually complex, parallel to main body axis and directed forward, but often either crossing each other or, more rarely, directed more or less laterad, dorsolaterad or, exceptionally, even caudad, also exceptionally perforating lateral walls of coxites. A solenomere, an exomere and/or an endomere often present, sometimes these being prominent. An accessory seminal chamber and/or a hairy pulvillus at its orifice only seldom developed, mostly tend to be absent.

##### Distribution.

All continents except Australia, mostly a Northern Hemisphere group only marginally represented in the Southern Hemisphere (Indonesia + New Guinea and Melanesia, Afrotropical realm north of Malawi and Madagascar, Neotropical realm north of Bolivia, Paraguay and southern Brazil), apparently fully allopatric with the superfamily Dalodesmoidea.

#### Family Trichopolydesmidae

Verhoeff, 1910

http://species-id.net/wiki/Trichopolydesmidae

##### Diagnosis.

Body polydesmoid, only exceptionally paraterga deeply lobulate laterally. Sphaerotrichomes only rarely present. Antennomere 5 sometimes devoid of sensilla distodorsally. Sphaerotrichomes only sometimes present.

Gonopod aperture large, transversely oval, with exposed to deeply sunken gonocoxae. Gonocoxae subglobose, usually with normal, tube-shaped cannulae, from small to rather large while telopodites from strongly exposed to concealed inside a considerable central gonocoel; prefemoral portion tends to be orientated transversely to main body axis, only occasionally somewhat to clearly shortened and thus resembling the condition observed in Polydesmoidea; acropodite tri-, bi- or uniramous, usually directed cephalad or cephalomesad; solenomere mostly evident, simple, only seldom a short tooth, more often long, distal in location, either stout or slender/flagelliform. Normally neither an accessory seminal chamber nor a hairy pulvillus, only exceptionally with a primordial accessory seminal chamber. *Caucasodesmus* Golovatch, 1985 (Caucasus and Crimea) is aberrant in having no sensilla on antennomeres 5 and no cannulae or seminal grooves.

Type genus: *Trichopolydesmus* Verhoeff, 1898.

##### Remarks.

The family Mastigonodesmidae Attems, 1914, based on *Mastigonodesmus* Silvestri, 1898 (ca 8 eight species in the western Mediterranean), is sometimes regarded as a synonym of Polydesmidae Leach, 1815 (Hoffman 1980, Simonsen 1990), apparently because the gonopod prefemoral part is shortened, but, due to globose gonocoxae and a peculiar, parabasal, long and coiled solenomere, it seems to be far more similar to that in trichopolydesmoids. The Mastigonodesmidae seems to also contain the monobasic genus *Ingurtidorgius*, which is sometimes treated as a subfamily of its own, because its male shows a peculiar hook on the mentum and totally suppressed lamellae linguales, coupled with a non-coiled, but flagelliform solenomere. Since the latter character is shared with *Trichopolydesmus*, contrary to some recent opinions (Mauriès 1983, Golovatch 2011), it seems best to also merge Mastigonodesmidae with Trichopolydesmidae, syn. n.

The same applies to the family Macrosternodesmidae, which basically fails to differ from Trichopolydesmidae, but simply tends to encompass quite a few genera with small to medium-sized, invariably globose gonocoxae and strongly exposed, often complex telopodites. Since the purely Nearctic nominate family Nearctodesmidae shares the gonopod conformation with Macrosternodesmidae, and it has sometimes been treated as only a subfamily or even a possible synonym of the latter family, I am inclined to treat these two latter families as synonyms of Trichopolydesmidae as well,syn. n.

Shelley (1994) gave a detailed morphological description of nearctodesmids and revived their family status, contrary to Hoffman (1982) who had synonymized the Nearctodesmidae with Macrosternodesmidae. Later, however, apparently following Shelley (1994), Hoffman (1999) also considered the Nearctodesmidae as a distinct family.

The diversity of gonopod structural plans in nearctodesmids+macrosternodesmids (Shelley 1994, Shear and Shelley 2007) appears to be quite modest and uniform, since all of their constituent genera such as *Nearctodesmus* Silvestri, 1910, *Kepolydesmus* Chamberlin, 1910, *Bistolodesmus* Shelley, 1994, *Tidesmus* Chamberlin, 1943 etc. show clearly transverse and elongated prefemoral portions which are set subrectangular to the subparallel, usually bi- or triramous, elaborate, normally clearly curved and well exposed acropodites. The gonocoel if any is moderate at most. According to Shear and Shelley (2007), the differences between these “families” lie only in the number and location of branches on the acropodite, a distinction that by far fails to exceed the diversity of gonopod plans observed among the South American fuhrmannodesmids alone (Golovatch 1994).

##### Contents and distribution.

Thus refined, the Trichopolydesmidae currently contains ca 20 genera and about 60 species in the Holarctic, as well as ca 55 genera and about 80 species in the tropics.

#### Family Opisotretidae

Hoffman, 1980

http://species-id.net/wiki/Opisotretidae

##### Diagnosis.

Sphaerotrichomes absent. Gonopod aperture large, transversely oval, with small and largely exposed coxites. Telopodites elongate, directed strongly dorsolaterad; seminal groove running along most of telopodite extent on caudal face to terminate distally either on a special branch or tooth (= solenomere), flush on caudal surface, or debauch inside an accessory seminal chamber which normally is supplied with a hairy pulvillus.

##### Type genus.

*Opisotretus* Attems, 1907

##### Contents and distribution.

Only seven genera and 29 described species ranging from the Ryukyu Islands, Japan, Taiwan, southern mainland China and the Himalayas in the North and Northwest, through Indochina and across Indonesia, to Papua New Guinea in the Southeast (Golovatch et al. 2013).

## Supplementary Material

XML Treatment for Galliocookia gracilis

XML Treatment for Sphaeroparia simplex

XML Treatment for Trichopolydesmoidea

XML Treatment for Trichopolydesmidae

XML Treatment for Opisotretidae

## References

[B1] Golovatch SI (1994) Further new Fuhrmannodesmidae from the environs of Manaus, Central Amazonia, Brazil, with a revision of *Cryptogonodesmus* Silvestri, 1898 (Diplopoda, Polydesmida). Amazoniana 13(1/2): 131–161.

[B2] Golovatch SI (2011) The millipede genus *Caucasodesmus* Golovatch, 1985, with the description of a new species from the Crimea, Ukraine (Polydesmida, Diplopoda, Trichopolydesmidae). ZooKeys 93: 1-8. doi: 10.3897/zookeys.93.1159 PMC3095180 21594076

[B3] Golovatch SI (in press) Three new species of the millipede genus *Hyleoglomeris* Verhoeff, 1910 from the Aegean region of Greece (Diplopoda, Glomerida, Glomeridae). Biodiversity Data Journal. 10.3897/BDJ.1.e1000 PMC3964707 24723748

[B4] Golovatch SI Gerlach J (2010) Class Diplopoda De Blainville in Gervais, 1844. In: Gerlach J Marusik Y (Eds) Arachnida and Myriapoda of the Seychelles islands. Siri Scientific Press, Manchester, 387-402.

[B5] Golovatch SI Geoffroy J-J Stoev P Vanden Spiegel D (2013) Review of the millipede family Opisotretidae (Diplopoda, Polydesmida), with descriptions of new species. ZooKeys 302: 13–77. doi: 10.3897/zookeys.302.5357 PMC3689142 23794898

[B6] Harvey MS (2008) Pseudoscorpions of the World, version 1.1. Western Australian Museum, Perth. http://www.museum.wa.gov.au/arachnids/pseudoscorpions/ [accessed 29.09.2013]

[B7] Hoffman RL (1980) Classification of the Diplopoda. Muséum d’histoire naturelle, Genève, 238 pp.

[B8] Hoffman RL (1982) Diplopoda. In: Parker SP (Ed) Synopsis and Classification of Living Organisms. McGraw-Hill Book Company, New York & St. Louis 2: 689–724.

[B9] Hoffman RL (1999) Checklist of the millipeds of North and Middle America. Virginia Museum of Natural History Special Publication Number 8: 1-584.

[B10] Mauriès JP (1983) Le genre *Galliocookia* Ribaut, 1954. Deux espèces nouvelles des grottes de l’Ardèche et du Gard (Myriapoda, Diplopoda, Polydesmida). Bulletin de la Société d’Histoire naturelle de Toulouse 119: 103-110.

[B11] Mauriès JP (1984) Deux espèces nouvelles de Diplopodes cavernicoles des Cyclades: *Hyleoglomeris beroni* (Glomerida) et *Syrioiulus andreevi* (Iulida). Biologia Gallo-Hellenica 11(1): 37–49.

[B12] Mauriès JP Geoffroy JJ (1999) Les Diplopodes édaphiques et souterrains de l’Ile Maurice (Myriapoda, Diplopoda). Revue suisse de Zoologie 106(1): 69-79.

[B13] Mauriès JP Heymer A (1996) Nouveaux micropolydesmides d’Afrique centrale: essai de rassemblement pour une révision du genre *Sphaeroparia* (Diplopoda, Polydesmida, Fuhrmannodesmidae). Bulletin du Muséum national d’Histoire naturelle, 4^e^ série, 18 (Section A, Nos 1-2): 165–184.

[B14] Schmalfuss H (2003) World catalog of terrestrial isopods (Isopoda Oniscidea). Stuttgarter Beiträge zur Naturkunde, Serie A, Nr 654, 341 pp.

[B15] Shear WA (2011) Class Diplopoda de Blainville in Gervais, 1844. In: Zhang ZQ (Ed) Animal biodiversity: An outline of higher-level classification and survey of taxonomic richness. Zootaxa 3148: 159–164. 10.11646/zootaxa.3703.1.1 26146682

[B16] Shear WA Shelley RM (2007) The milliped genus *Tidesmus* Chamberlin, 1943 (Polydesmida: Macrosternodesmidae). Zootaxa 1656: 51-68.

[B17] Shelley RM (1994) The milliped family Nearctodesmidae in northwestern North America, with accounts of *Sakophallus* and *S. simplex* (Chamberlin) (Polydesmida). Canadian Journal of Zoology 72: 470-495. doi: 10.1139/z94-066

[B18] Simonsen Ǻ (1990) Phylogeny and biogeography of the millipede order Polydesmida, with special emphasis on the suborder Polydesmidea. Museum of Zoology, University of Bergen, 114 pp.

